# Building a National Reassessment Process for Oncology Drugs: Lessons Learned by the Canadian Real-World Evidence for Value of Cancer Drugs (CanREValue) Collaboration through a Simulated Reassessment Exercise

**DOI:** 10.3390/curroncol28060392

**Published:** 2021-11-12

**Authors:** Wei Fang Dai, Erica Craig, Brent Fraser, Alex Chambers, Helen Mai, M. Bryson Brown, Craig C. Earle, William K. Evans, Marc Geirnaert, Marianne Taylor, Maureen Trudeau, Daniel Sperber, Jaclyn M. Beca, Avram Denburg, Rebecca E. Mercer, Ambica Parmar, Mina Tadrous, Pam Takhar, Kelvin K. W. Chan, on behalf of the CanREValue Collaboration

**Affiliations:** 1 Temerty Faculty of Medicine, University of Toronto, Toronto, ON M5S 1A8, Canada; weifang.dai@mail.utoronto.ca; 2 Canadian Centre for Applied Research in Cancer Control, Toronto, ON M5G 2L3, Canada; Jaclyn.Beca@ontariohealth.ca (J.M.B.); rebecca.mercer@ontariohealth.ca (R.E.M.); 3 New Brunswick Cancer Network, Fredericton, NB E3B 5G8, Canada; Erica.Craig@gnb.ca; 4 Canadian Agency for Drugs and Technologies in Health, Ottawa, ON K1S 5S8, Canada; BrentF@cadth.ca (B.F.); alex.chambers@rogers.com (A.C.); helenm@cadth.ca (H.M.); 5 Philosophy Department, University of Lethbridge, Lethbridge, AB T1K 3M4, Canada; brown@uleth.ca; 6 Sunnybrook Health Sciences Centre, Toronto, ON M4N 3M5, Canada; craig.earle@ices.on.ca (C.C.E.); maureen.trudeau@sunnybrook.ca (M.T.); ambika.parmar@sunnybrook.ca (A.P.); 7 Department of Oncology, McMaster University, Hamilton, ON L8S 4L8, Canada; billevans@cogeco.ca; 8 Ontario Health (CCO), Toronto, ON M5G 2L3, Canada; pam.takhar@ontariohealth.ca; 9 CancerCare Manitoba, Winnipeg, MB R3E 0V9, Canada; mgeirnaert@cancercare.mb.ca; 10 BC Cancer, Kelowna, BC V1Y 5L3, Canada; mtaylor@bccancer.bc.ca; 11 Pan-Canadian Pharmaceutical Alliance, Toronto, ON M5S 2B1, Canada; Daniel.Sperber@ontario.ca; 12 The Hospital for Sick Children, Toronto, ON M5G 1X8, Canada; avram.denburg@sickkids.ca; 13 Women’s College Hospital, Toronto, ON M53 1B2, Canada; Mina.Tadrous@wchospital.ca; 14 Leslie Dan Faculty of Pharmacy, University of Toronto, Toronto, ON M53 3M2, Canada

**Keywords:** real-world evidence, health technology assessment, reassessment

## Abstract

The CanREValue Collaboration established the Reassessment & Uptake Working Group to develop a preliminary process to reassess funded cancer drugs in Canada. A simulated exercise was conducted to evaluate the proposed reassessment process using a real-world case. We invited 32 attendees including representatives from Health Canada and Health Technology Assessment (HTA) agencies, along with payers, clinicians, academics, and patient representatives. A case was developed using a real-world study on a publicly funded cancer drug. In facilitated group sessions, participants were asked to deliberate upon the evidence presented in the case to issue reassessment recommendations. Several themes were identified through the deliberation discussions. While the generalizability of real-world evidence (RWE) is perceived as a strength, trust in the RWE depends largely on the source of the real-world data. The attendees suggested several improvements to the proposed reassessment process including evidence requirement for reassessment, recommendation categories, and a priori study protocols. This exercise generated important insights on the evidence required for conducting reassessment and considerations for improvements of the proposed reassessment process. Building upon lessons from this exercise, future work would continue to refine the reassessment process as part of the overall CanREValue framework.

## 1. Introduction

A new, internationally accepted definition of health technology assessment (HTA) was recently developed by the International Network of Agencies for Health Technology Assessment and Health Technology Assessment International [1,2] to broaden the scope of HTA to encompass lifecycle health technology management (HTM). An HTM approach focuses on assessing and managing the value of a health technology at different points in its lifecycle, from pre- to post-market, to inform decision-making on disinvestment, refine reimbursement criteria, and influence price re-negotiation [2]. Central to the HTM approach is the need to collect relevant and up-to-date evidence for the purpose of re-evaluating and re-assessing the benefit and impact of a health technology.

The reassessment of health technologies can inform their optimal use, with the aim of improving the quality of clinical care while ensuring the sustainability of healthcare systems [3,4]. In therapeutic areas such as oncology, where treatment options are rapidly evolving and expenditures more than doubled from 2005 to 2012 in Canada [5], reassessment of a funded treatment is particularly valuable as it could inform the optimal allocation of healthcare resources [5]. After a treatment has been funded, real-world data (RWD) could be collected and real-world evidence (RWE) about the benefits, safety and value of the treatment can be generated [6]. Because RWE is derived from clinical practice, it can describe the impact of treatment in patients who are often excluded from clinical trials [7]. The RWE generated can then be used to inform decisions about continued drug funding and assist with price re-negotiation [8,9,10].

The Canadian Real-world Evidence for Value in Cancer Drugs (CanREValue) Collaboration was established in 2017, consisting of members from drug approval regulators, pricing regulators, HTA agencies representatives, pan-Canadian price negotiators, HTA committee members, public payers, clinicians and patient representatives. As part of the overall objective to develop a framework for the generation and use of RWE to inform cancer drug funding decisions [11], the CanREValue collaboration established the RWE Reassessment and Uptake Working Group (Reassessment WG). The Reassessment WG has since developed a proposed reassessment process that uses RWE [12]. Modelled after the existing initial assessment process used at the Canadian Agency for Drugs and Technologies in Health (CADTH) [13], the Reassessment WG recommended that a reassessment submission file, with new evidence on a funded cancer drug, be reviewed and evaluated by a multidisciplinary committee. After deliberating on the new evidence, the committee would issue one of three recommendations: maintain status quo, revisit funding criteria or pricing, or do not continue funding/consider disinvestment strategies [12].

To evaluate the proposed reassessment process developed by the Reassessment WG, the CanREValue Collaboration conducted a simulation exercise. A real-world case study was developed using a funded cancer drug, bevacizumab for metastatic colorectal cancer, to illustrate a potential reassessment submission file [14,15,16]. Members of the CanREValue Collaboration were convened to review the case study, deliberate upon the RWE, issue a reassessment recommendation, and critique the proposed process. This paper outlines the main lessons learned from the simulation exercise with the intent to inform future refinement and improvement of the reassessment process by the Reassessment WG.

## 2. Approach

### 2.1. Study Context

On 29 May 2019, 32 members of the CanREValue Collaboration convened in Halifax, Canada to conduct a simulation exercise. The aim of the exercise was to evaluate the proposed reassessment process developed by the Reassessment WG. Relevant backgrounds of the attendees are listed in Table 1. Since some attendees are affiliated with multiple organizations, they have been listed under more than one of the background categories.

### 2.2. Simulation Exercise Format

The format of the simulation exercise was adapted from the deliberative process currently used by CADTH, the pan-Canadian Oncology Drug Review Expert Review Committee (CADTH-pERC), during the initial assessment of oncology drugs for funding. The half-day simulation exercise consisted of three sections: (1) presentation of the proposed framework and case study; (2) facilitated breakout group discussion (8–9 attendees); and (3) large group discussion (Table 2). The case study and facilitator guide were shared with attendees and facilitators of the breakout groups, respectively, one week before the simulation exercise (Appendix A).

The first section included a presentation of the proposed reassessment process by the chair of the Reassessment WG and the case study by four experts. The case study was developed from a real-world evaluation of bevacizumab, a publicly funded cancer drug for metastatic colorectal cancer, conducted in three Canadian provinces—Ontario, British Columbia, and Saskatchewan [14,15,16]. The real-world evidence on bevacizumab was presented alongside the initial randomized controlled trials (RCT) results reviewed during the initial HTA evaluation. The clinical evidence was presented by a clinician. The economic evidence was presented by an academic clinician with economic experience. The patient value evidence was presented by a patient representative. The implementation and sustainability evidence was presented by a provincial cancer agency decision-maker. A facilitator guide was also developed to assist the facilitators when leading the breakout group sessions.

Following the presentation, attendees were divided into breakout groups led by facilitators. In each group, the facilitators led a deliberation on the new RWE evidence with the aim of reaching a consensus within the group on a reassessment recommendation, as well as gather feedback and critique on the proposed reassessment process. All attendees then convened to share their reassessment recommendations and debrief their experience.

Each breakout group was assigned a scribe to take notes using a standardized template. The notes were summarized and main themes from the discussions were synthesized and shared with the attendees within a month after the in-person simulation exercise.

## 3. Findings

The main themes that emerged from the attendees’ discussions are described below.

### 3.1. Generalizability of Real-World Evidence Is Perceived as a Strength

Attendees felt that it was a major strength that the results of the real-world case study could be generalized to the larger Canadian population. Specifically, attendees noted that having RWE about clinical benefit and cost-effectiveness from multiple provinces instills confidence in the results, and that this is particularly useful in the context of a reassessment process intended to support policy decisions. RWE derived from single institutions were not considered as credible as those derived from more diverse populations due to their narrow focus, lack of generalizability, and potential for bias. It is important to note that some attendees also discussed the possibility of using RWE from other jurisdictions and even international sources to supplement the decision-making process in their own jurisdiction. Attendees stated that, when data collection methods are aligned with accepted standards and potential biases are addressed by methodology experts, they would be open to using RWE from other jurisdictions to inform decision-making. In some contexts such as rare disease, attendees acknowledged the difficulty of reaching the necessary sample size to conduct a robust real-world study based only on Canadian data, thus making the use of RWE from other jurisdictions still more appealing.

### 3.2. Trust in Real-World Evidence Pertaining to Patient Value Depends on the Source of the Real-World Data

Attendees believed evidence of patient values and experiences were crucial in the reassessment of funding decisions. In this case study, data on patient values presented for reassessment were obtained from a literature review of published results from international sources and did not come from the Canadian population. As a result, attendees were uncertain whether the evidence adequately reflects Canadian patient values. Moreover, the patient experience evidence was obtained from a small sample, so attendees were uncertain about the robustness of the results. In light of these issues, some attendees struggled with issuing a reassessment recommendation because of the lack of appropriate data reflecting the patient experience. Attendees recognized and discussed how trust and confidence in RWE depends on the sources of data and methods of data collection. Transparency around data sources and critical appraisal of RWE were also highly valued. Attendees also emphasized that when conducting RWE for the purpose of reassessment, the source of databases and/or the method of collecting data should align with existing standards or guidelines, to ensure that the evidence presented could be interpreted with confidence.

### 3.3. Establishing a Cost-Effectiveness Threshold

When reviewing the economic evidence, some attendees suggested that an explicit cost-effectiveness threshold could benefit the reassessment review, especially when the incremental cost-effectiveness ratio (ICER) from the real-world study is different from the ICER used for initial funding approval. Other attendees were hesitant to establish an explicit cost-effectiveness threshold for reassessment as there is no currently existing, formally accepted threshold to guide initial HTA recommendations in Canada, which is similar to the process in France but unlike that of the United Kingdom [17]. Those who favored the idea of establishing an explicit cost-effectiveness threshold noted that operationalizing a threshold for economic evidence in the absence of such a threshold during initial assessment can be challenging. Some attendees suggested that, if a cost-effectiveness threshold were to be set for the purpose of reassessment, there should be strict adherence whereby only drugs that fall below the threshold would be issued a recommendation for “remain at status quo” and maintenance of the current reimbursement recommendation. In the context of reassessment, the evidence submitted for evaluation should have less uncertainty compared to the initial submission, therefore strict adherence to the cost-effectiveness thresholds should be used to support continued funding. Otherwise, for drugs that exceed the cost-effectiveness threshold based on RWE, price re-negotiation should be recommended, provided there are no concerns with respect to magnitude of clinical benefits and safety in the real-world. In light of the ongoing considerations by the Patented Medicine Prices Review Board, a national independent quasi-regulatory body, regarding the establishment of an initial value threshold when evaluating the economic evidence high-cost medicines, the issue around cost-effectiveness thresholds will need to be considered for future iterations of the reassessment process [18].

### 3.4. Collecting Relevant Evidence to Inform Reassessment

The breakout group attendees identified additional evidence that was not included in the case study but would be required to conduct a satisfactory reassessment (Table 3). Attendees emphasized the importance of collecting data directly from patients, regarding their experiences and expectations, such as quality of life and patient-reported outcomes, to understand whether the treatment aligned with patient values in the real-world context. Attendees also noted the importance of having real-world economic data that were comparable to the economic evidence considered during the initial HTA. In the case study, economic data were presented in terms of “life-years gained” due to the lack of real-world utility values. Initial drug funding assessments typically review quality-adjusted life years (QALYs) using utility values collected during the clinical trials. While the attendees acknowledged that utility values are not currently collected in population-based administrative datasets, they felt QALY calculations are important for evidence comparison. Some attendees suggested using utility values from published literature to estimate real-world QALYs in the absence of real-world utility values. Finally, the attendees acknowledged that some of the additional evidence proposed is not currently available in population-based databases. Potential approaches to gathering these data were suggested, including obtaining access to data from patient support programs sponsored by the pharmaceutical industry or establishing data collection in the population-based administrative databases following initial funding. Some attendees noted that accessing data from patient support programs might be complicated by privacy issues including the need to obtain patient consent, while other attendees expressed concerns about relying on corporate data collection and management.

### 3.5. Patient Engagement

Patient engagement has been considered essential throughout the development of the reassessment process. During discussions, attendees emphasized that patients should be consulted and included throughout the process of reassessment. Attendees suggested that patient engagement could include using RWD about patient experiences and expectations as part of the reassessment. Additional suggestions included reaching out to patient groups with surveys or questionnaires, and/or working with physicians and researchers to collect patient reported outcomes from available databases. It was noted that transparency was essential and that patients need to be kept informed about the process and made aware that reassessment could potentially lead to restrictions in access to some treatment options and to defunding of others. Attendees emphasized that patients should have the opportunity to provide their perspective on potential trade-offs in resource allocations if existing drug funding decisions are modified. Some attendees pointed out that a deliberative approach coupled with multi-criteria decision analysis has been successful for other HTA agencies when engaging with patients. Lastly, attendees recommended that the same patients/patient representative groups involved in the initial review should be included when a drug is undergoing reassessment.

### 3.6. Modifying Recommendation Categories

A concern was raised about the proposed recommendation categories of (1) status quo (i.e., continue funding), (2) revisit funding criteria or pricing, or (3) do not continue funding/delist. After reviewing and deliberating upon the RWE within the four breakout groups, attendees’ opinions were divided between remain at status quo and revisit funding or pricing. The attendees felt that the recommendation categories limited their ability to convey the nuances and context of their deliberation. Some attendees suggested that the proposed categories should resemble recommendations that would have been issued by a pricing body or payer instead of an HTA agency. Further, it was suggested that the recommendation categories could be refined to focus on the new evidence presented and how it led to the recommendation. Others also suggested separating the second criteria into two explicit categories: revisiting funding criteria and revisiting pricing.

### 3.7. Accounting for Context and Changing Landscape

Attendees emphasized that the cancer treatment landscape is rapidly evolving. When undertaking reassessment reviews, there will be a need to take into consideration this rapidly changing therapeutic landscape. For example, the introduction of biosimilars or changes in treatment sequencing as a result of new treatment options will have to be taken into consideration. It will, therefore, be important to consider how the reassessment recommendation on a funded drug might influence and/or be influenced by other drugs new to the therapeutic space. For these reasons, the timing of a reassessment review could be critical, and reassessment should be done based on the needs of payers while also taking account of expected changes in the treatment landscape.

### 3.8. A Priori Study Plan

Attendees felt that it was important to develop an a priori standard for conducting RWE analyses. This study plan would include, but not be limited to, study design, outcomes, sample size, and follow-up period. Attendees also suggested establishing predefined outcome thresholds that could lead to disinvestment during reassessment; however, they acknowledged that it could be difficult to determine these thresholds. Lastly, attendees emphasized that the development of an a priori study plan must be agreed upon by all relevant stakeholders, including industry representatives.

## 4. Discussion

In this simulation exercise, we evaluated a proposed reassessment process developed by the CanREValue Collaboration using a case study developed from a real-world study of bevacizumab for metastatic colorectal cancer. This exercise identified some key learnings and useful insights that can be used to refine the proposed reassessment process. While previous literature has proposed various reassessment frameworks, much of this work has been either theoretical or based on key informant interviews [3,4,19]. In 2013, MacKean et al. conducted a workshop which identified several main ideas that can advance the field of health technology reassessment including a call to focus on the development of reassessment methods and processes as well as meaningful stakeholder development. In 2017, Soril et al. proposed a conceptual reassessment model that includes a decision phase with both evidence synthesis and policy recommendation steps [4], which is similar to the reassessment process proposed by the CanREValue Collaboration. Building upon the lessons from the previous work, we convened a group of key stakeholders to evaluate a proposed reassessment process through a simulation exercise to tailor to the context in Canada. The findings from our simulation exercise complements the existing work and provides additional insights and considerations about the kinds of evidence (e.g., performance status, patient-reported outcomes, and quality-adjusted life-years) required when conducting reassessments in the Canadian context based on inputs from Canadian stakeholders specifically.

Insights from this simulation exercise may be valuable to international HTA agencies that have adopted, or are in the process of adopting, reassessment processes [20,21,22,23,24,25,26]. Since 2004, new drugs approved by the Health Ministry in France received a registration for reimbursement for 5 years, after which drugs are systematically re-evaluated by the Haute Authorite de Sante (HAS) [25]. In Brazil, the National Committee for Health Technology Incorporation (Conitec) was established in 2011 with the mandate to advise the Ministry of Health regarding the adoption and/or disinvestment of health technologies [20]. As part of reassessment, Conitec considers scientific evidence including efficacy, effectiveness, safety, budget impact and economic analyses. In 2006, the National Institute of Health and Clinical Excellence (NICE) in the UK attempted to pilot the ineffective treatments program to appraise health procedures in order to identify those of low value that may be suitable for disinvestment; however, it was later felt that this program was not necessary as NICE has been consistently producing “do not do” recommendations in collaboration with Cochrane Review during the existing HTA process [25]. Finally, several countries in Asia are also refining and/or developing HTA processes which include exploring reassessment procedures and developing frameworks to integrate reassessment into the HTA processes [21,23]. In Canada, CADTH has outlined a reassessment process in its recent procedural update and the lessons from this simulation exercise may inform the future refinement of CADTH’s reassessment processes [13].

## 5. Conclusions

The CanREValue Collaboration conducted a simulation exercise to evaluate a proposed reassessment framework. Multiple attendees across Canada representing diverse perspectives were convened to reassess real-world evidence on a publicly funded drug, bevacizumab, for metastatic colorectal cancer. This simulation exercise identified several key considerations for potential improvements of the proposed reassessment process. Future work of the CanREValue Collaboration will focus on developing a framework that will enable the integration of reassessment recommendations into drug funding policy decisions. As the CanREValue Collaboration continues to develop the framework for life-cycle health technology management, further consultations will be conducted to ensure that the framework is comprehensive and addresses the needs of all relevant stakeholders.

## Figures and Tables

**Table 1:** Attendee representative list.

Representative	Sample Size
Health Canada representatives	N = 2
PMPRB representatives	N = 2
pCPA representatives	N = 2
Clinicians—Medical Oncologists	N = 7
CADTH/INESSS Staff	N = 5
pERC Committee members (Current or Previous)	N = 6
Public Drug Plan Administrators	N = 8
Patient representatives	N = 2
Academics	N = 6

Note: Some attendees (n = 8) represent more than one perspective. Abbreviations: CADTH = Canadian Agency for Drugs and Technologies in Health; INESSS = Institute national d’excellence en santé et en services sociaux (INESSS); pCPA = pan-Canadian Pharmaceutical Alliance; pERC = pan-Canadian Oncology Drug Review Expert Review Committee. PMPRB = Patented Medicine Prices Review Board.

**Table 2:** Simulation exercise format.

Agenda	Content
Proposed reassessment process	Introduce the proposed reassessment process
Case-study: bevacizumab for metastatic colorectal cancer	Clinical Evidence: RWE on overall survival and safety data
	Economic evidence: RWE on average cost per patient over time and the incremental cost-effectiveness ratio (ICER)
	Patient value evidence Patient-reported symptoms and experiences from published literature
	Implementation and Sustainability evidence RWE on budget impact
Breakout Group Discussion	Deliberate new evidence on the funded drug Issue a reassessment recommendation Critique the reassessment process
Group discussion	Debrief

**Table 3:** Proposed additional evidence that should be included in the reassessment process.

Evidence Categories	Elements
Clinical evidence	Performance status Dose delays and dose intensity Long-term toxicity Sub-groups that may benefit less from treatments
Patient values	Patient experiences and expectations Patient-reported outcomes Caregiver inputs Quality of Life (QoL) collected using validated tools
Economic evidence	Quality adjusted life years Utility values ICER using confidential prices
Implementation & Feasibility	Disease-specific treatment maps to describe current treatment sequencing and how sequencing might change dependent of the outcome of the reassessment Resource reallocation: where would the resources be reallocated from?

Abbreviations: ICER, incremental cost-effectiveness ratio; QoL, Quality of Life.

## Data Availability

The data that support the findings of this study are available upon request from the corresponding author.

## Appendix A. Case Study (Abridged)

### Appendix A.1. Background

When a new oncology drug is recommended for public funding by the pCODR expert review committee, the evidence is largely based on those from available trials. However, the results observed from trials may not reflect the real world. After a drug is publicly funded, real-world data can be collected to evaluate the real-world benefit. As such, there needs to be processes in place to help reassess the initial recommendation.

### Appendix A.2. Mock Reassessment

**Objective 1:** You will be presented real-world evidence from a real-world study. With this evidence, you will undergo a mock reassessment process to assess the clinical evidence, patient evidence, cost-effectiveness, and implementation and sustainability of a cancer drug to make one of the following recommendations:

- Remain at status quo
  ⚬ The provided evidence confirmed the effectiveness, safety, and cost-effectiveness. There is no need to change the current reimbursement recommendation.
  ⚬ The provided evidence was insufficient to address an important question of effectiveness, safety, or cost-effectiveness. More data are required.
- Revisit funding criteria or pricing
  ⚬ Based on the new evidence, funding criteria need to be revised (i.e., broader or narrower indication)
  ⚬ Based on the new evidence reviewed, the cost effectiveness of the drug has changed. Jurisdictions should evaluate whether existing pricing arrangements reflect the revised cost effectiveness.
- Do not continue to fund/delist
  ⚬ Based on the new evidence, the committee concluded that there was at least one better alternative treatment available.
  ⚬ Based on the new evidence, the committee concluded that there was a potential safety concern.

**Objective 2:** In prior surveys, the members have recommended the following criteria for reassessment: clinical evidence, patient evidence, cost-effectiveness, and implementation and sustainability. You will evaluate the current reassessment process and the four criteria.
